# An Overview of the Control of Bacterial Pathogens in Cattle Manure

**DOI:** 10.3390/ijerph13090843

**Published:** 2016-08-25

**Authors:** Christy E. Manyi-Loh, Sampson N. Mamphweli, Edson L. Meyer, Golden Makaka, Michael Simon, Anthony I. Okoh

**Affiliations:** 1 Fort Hare Institute of Technology, University of Fort Hare, Alice Campus, Alice 5700, Eastern Cape Province, South Africa; smamphweli@ufh.ac.za (S.N.M.); emeyer@ufh.ac.za (E.L.M.); msimon@ufh.ac.za (M.S.); 2 Applied and Environmental Microbiology Research Group (AEMREG), Department of Biochemistry and Microbiology, University of Fort Hare, Alice Campus, Alice 5700, Eastern Cape Province, South Africa; aokoh@ufh.ac.za; 3 Department of Physics, University of Fort Hare, Alice Campus, Alice 5700, Eastern Cape Province, South Africa; gmakaka@ufh.ac.za; 4 South Africa Medical Research Council Microbial Water Quality Monitoring Centre, University of Fort Hare, Alice 5700, Eastern Cape Province, South Africa

**Keywords:** cattle manure, pathogens, control methods

## Abstract

Cattle manure harbors microbial constituents that make it a potential source of pollution in the environment and infections in humans. Knowledge of, and microbial assessment of, manure is crucial in a bid to prevent public health and environmental hazards through the development of better management practices and policies that should govern manure handling. Physical, chemical and biological methods to reduce pathogen population in manure do exist, but are faced with challenges such as cost, odor pollution, green house gas emission, etc. Consequently, anaerobic digestion of animal manure is currently one of the most widely used treatment method that can help to salvage the above-mentioned adverse effects and in addition, produces biogas that can serve as an alternative/complementary source of energy. However, this method has to be monitored closely as it could be fraught with challenges during operation, caused by the inherent characteristics of the manure. In addition, to further reduce bacterial pathogens to a significant level, anaerobic digestion can be combined with other methods such as thermal, aerobic and physical methods. In this paper, we review the bacterial composition of cattle manure as well as methods engaged in the control of pathogenic microbes present in manure and recommendations that need to be respected and implemented in order to prevent microbial contamination of the environment, animals and humans.

## 1. Introduction

Livestock industries are growing tremendously along with the human population ensuing in the increase rate of generation of organic residues, which pose problems that warrant strategies for disposal and/or management [1]. Agricultural animals, including cattle produce copious quantities of animal manure consisting of animal excreta (feces and urine) along with bedding, microorganisms, process-generated wastewater, secretions (nose, throat, blood, vagina, mammary glands, skin and placenta), undigested and spilled feed, antibiotics, nutrients and fur [2]. In addition, animal manure is known to harbor a wide variety of microorganisms which can be pathogenic or non-pathogenic to both animals and humans [3]. The levels and types of pathogens occurring in livestock wastes vary with animal species, dietary sources, health status and age of the animals, physical and chemical characteristics of the manure produced as well as the storage facilities of the manure [4,5].

Clearly, the pathogenic microbes enumerated so far in livestock wastes entail bacteria, viruses and protozoa. However, owing to differences in microbial characteristics between and within these groups of organisms, the extent to which they survive in the environment or manure depends fundamentally on the characteristics of the particular organism, the source and chemical composition of manure (e.g., ammonium content), pH, dry matter, temperature, oxygen, microbial competition and moisture of these materials [6,7,8]. It is therefore apparent that these wastes have to be properly contained and managed as they can cause infections in animals and in humans either through direct contact with animals or animal wastes or through contaminated food or water.

Furthermore, it is imperative that adequate multiple management interventions should be instituted backed by the local, social and economic status of the farms and also regulatory guidelines. This is because of the different survival rates of the pathogens in a bid to significantly reduce pathogens in these wastes to levels recommended for public health safety. This is described as the microbiological quality of the treated wastes and relies on the level of residual microorganisms after treatment. However, the microbial level of the treated wastes is dependent on the type of treatment [9] and the acceptable threshold levels of the known pathogens of public health significance vary between countries due to the differences in regulations/guidelines [10,11]. Taking into consideration composting as the treatment process, the hygienic standard of compost varies from country to country and even within states and local governments owing to the scope and complexity of guidelines as is the case with USA. All the same, these can be represented as shown in Table 1.

In this regard, Spiehs and Goyal [5] recommended that the best management practices to reduce pathogens in livestock wastes should be approached from three angles: reducing pathogens in the animals, during manure collection and storage and lastly during land application of manure. Contrarily, raw manure and improperly treated manure can serve as a source of pathogenic contamination causing soil, air and water pollution, which in turn will result to critical public health issues. The devastating outcomes of the deliberation or accidental release of animal wastes into the environment have been delineated by other authors as well [3,6,16].

Notwithstanding, depending on the size of the farm and economic feasibility, biological, chemical and physical methods may be employed to reduce the pathogen level of the animal wastes before application on land for soil amendment [17,18]. It is worth mentioning that even a small reduction in the levels or the occurrence of these pathogens will be significant because usually large quantities of these wastes are generated. Therefore, it will lower the risk of pathogen transfer from manure during land application [4]. Accordingly, numerous studies conducted around the world investigated the fate of bacterial pathogens in animal manure during different treatment methods. For instance, Hutchison et al. [4] analyzed stored waste manures of different ages for the levels of zoonotic pathogens, but noted no significant difference in the level of the pathogenic bacteria irrespective of the duration of storage. Contrarily, Simujide et al. [19] demonstrated that food borne pathogens were significantly reduced in cattle manure during mesophilic composting, with the addition of 2.5% calcium cyanide. However, during composting the green house gas, carbon dioxide is released into the environment causing global warming [20].

To address these identified weaknesses or shortcomings of the other available methods employed for the treatment of animal wastes, anaerobic digestion of cattle manure in an airtight chamber has been presented as a promising method to restrain the emission of the green house gases and also reduce the pathogenic level to a recommendable point for public health safety. In addition, this technique results in the stabilization of wastes producing biogas (a renewable energy), improving the fertilization efficiency of treated waste to be used as a soil conditioner and combats the issue of nuisance from odors and flies [21].

Consequently, this paper describes the bacterial composition of cattle manure, the factors affecting bacterial survival rates, the methods engaged in the control of pathogenic microbes present in manure, the influence of the final products, compost and digestate (derived from composting and anaerobic digestion, respectively) on pathogenic load upon soil application and to end with, the recommendations that need to be respected and implemented in order to prevent microbial contamination of the environment, animals and humans.

## 2. Microbial Composition of Cattle Manure

Literally, livestock practices vary from one individual to another and from one geographical location to the other, but eventually, influence the microbial structure of manure released by the animals. Clearly, manure provides different biological and physicochemical environment to microorganisms [22]. Specifically, Pachepsky et al. [23] noted that many manure-based pathogens exist, but the major manure based zoonotic bacteria, including *Salmonella* spp., *Campylobacter* spp., *Listeria monocytogenes*, *Yersinia* *enterocolitica*, *Escherichia coli* and protozoa viz. *Cryptosporidium parvum* and *Giardia lamblia*, are present; however others are less common. Viruses represent another group of pathogens that exist in cattle manure. Originally, these pathogens inhabit the intestinal tracts of animals and are typically shed in this habitat asymptomatically [24]. Seemingly, both animals and humans on and off farms are exposed to the potential health risks allied to inadequate management of manure. Consequently, the fate of these pathogens in manure to pollute, contaminate and infect the environment and humans, respectively, is based on the pathogen’s ability to survive in manure following excretion [22]. Nevertheless, the factors affecting the survival rates of these well documented pathogens excreted in cattle manure have been enumerated and deliberated on as we proceed through this paper.

The dissemination of these pathogens could occur via unplanned and uncontrolled release by runoff either from livestock facilities or excessive land application of manure and also through infiltration into soils and groundwater or by the release of biosolids and manure residuals upon transportation off farms in circumstances where land application, marketing or other beneficial uses are appropriate [5,22]. It is worth mentioning that vectors, e.g., flies and vermin, may also spread and cause subsequent infections to other animals with pathogens from stored manure. The microbes presented below were chosen based on their probability of dissemination from cattle manure to humans as well as their endorsed potential health threats to humans and the environment. Moreover, outbreaks caused by these organisms have been linked tenuously to cattle in certain instances [6]. Table 2 shows the different pathogen types, their prevalence, storage condition and survival periods in shed cattle manure after pollution, transportation and infection, as well as the diseases/symptoms they cause in humans.

### 2.1. Classification and Description of the Main Bacterial Pathogens in Cattle Manure

It is quite unrealistic to enumerate all the microbial pathogens present in cattle manure because of the huge numbers of these pathogens that populate the gastrointestinal tract and the other systems in the animal. Diverse groups of microbial pathogens are involved due to the vast array of physical, chemical and biological constituents contained in cattle manure [16]. However, it is impossible for every individual pathogen that constitute the entire bacterial organization in manure to be isolated, characterized and identified [33]. These challenges may be linked to the limitations in some of the available methods failing to identify some of these pathogens owing to the state they may be exhibiting at that particular instance. In addition, differences in the DNA extraction and purification methods can affect DNA quality, which in due course can affect the interpretation of information regarding the microbial communities coexisting in manure samples [34,35].

Furthermore, some pathogens may require time-intensive tests and enrichment steps during analysis and detection, thereby making their quantification complex [36]; consequently, the pathogens represented herein, are limited to those considered as normal flora in the gut of these animals and those excreted in infections of the respiratory, urinary, and genital organs. In addition, the odds of transmission of these pathogens to humans and their would-be pressure to cause infections and diseases in animals and humans are well recognized in animal farming in times past [6].

However, the possibility of causing an infection depends on the type of bacteria, its infective dose and the immune status of the individual host. Owing to the variation in pathogenic/virulence potential across the bacterial domain, the number of cells necessary to cause infection equally differs from one bacterium to the other. This indicates that as few as 10 cells of a specific bacterium may be sufficient to cause an infection, whereas a greater number of cells may be required by others to cause an infection. However, small numbers of some bacteria shed in manure and introduced into the environment might multiply under favorable conditions resulting in higher levels, i.e., greater risk, thereby causing contamination of food, soil and water [25].

*Escherichia coli* is a gram-negative, facultative anaerobic, rod-shaped and motile bacterium that exists as a normal flora in the intestinal tract of both healthy animals and humans. It serves as a reliable indicator of fecal contamination and equally indicates a possibility of the occurrence of enteropathogenic and/or toxigenic microbes found in food and water, thereby presenting with public health hazards [37]. *E. coli* O157:H7 found in cattle manure has been reported as the most notorious pathogen which produces a potent toxin that can cause serious infection in humans. This strain can be called verocytotoxic (VTEC) or enterohemorrhagic (EHEC) or Shigatoxin producing (STEC) *E. coli*. According to Gerba and Smith [17], owing to the pathogen’s low infective dose ascribed to as few as 10 cells and high pathogenicity, it is incriminated as the causative agent of gastrointestinal diseases (gastroenteritis). In addition, Karmali [38] highlighted that the bacterium equally causes attaching and effacing (A/E) lesions by translocating effector proteins into the host cells via a type III protein secretion system. However, cattle harboring this *E*. *coli* strain do not develop clinical disease, but serve as the main reservoir for *E. coli* O157:H7 [39]. Furthermore, humans may be infected with pathogenic *E. coli* by way of either consuming contaminated food or water or directly by contact with livestock feces and person to person transfer [40]. In adverse/unfavorable environmental conditions (e.g., starvation), the bacterium alters its physiological or metabolic responses in order to persist in the environment without necessarily modifying its structure like most Gram positive bacteria that form resistant spores [26]. In addition, it is acid resistant and can equally form biofilm [41,42].

*Salmonella* species belong to the family of *Enterobacteriaceae* and are Gram negative, short, plump shaped rods, non-spore forming, motile, non-capsulated, aerobic and facultative anaerobic organisms [43]. These pathogens could be found in a range of animals, including dogs, birds, cats, cattle, pigs and humans and are responsible for the infection called salmonellosis. Salmonellosis occurs via ingestion of food or water contaminated with animal feces or by direct contact with animal feces and is characterized by three major symptoms viz. septicemia, acute enteritis and chronic enteritis [44]. However, the manifestations of salmonellosis vary depending on the species/strain and the host type [45].

*Salmonella* species viz. S. Dublin, S. Typhimurium and S. Newport constitute a major public health issue since they cause infections in both cattle and humans [27,46]. More elaborately, *Salmonellae* have developed increasing resistance to a wide spectrum of antibiotics (i.e., multidrug resistance, MDR) traditionally employed in the treatment of salmonellosis. These MDR strains are vital because of their resistance to available antibiotics, which are critical in the treatment of bacterial infections. However, the resistance could result in high death rates and also present an opportunity for epidemic outbreaks that may be difficult to manage [47].

*Campylobacter* species are most commonly found in the intestinal tracts of animals such as cattle, pigs, chickens, wild-living mammals and birds [48]. They are known to cause a wide variety of disorders in cattle, sheep, and pigs. For example, Salihu and coworkers [49] mentioned that *Campylobacter jejuni* and *Campylobacter fetus* caused abortion, stillbirths and birth of weak lambs in sheep at some stage in late pregnancy. Similarly, when transmitted to humans when consuming undercooked agricultural food products and contaminated water [50], it causes an infection called campylobacteriosis, which is a self-limited and sporadic illness [51,52]. In addition, *C*. *jejuni* and *Campylobacter* *coli* are the two most important species associated with human bacterial gastroenteritis worldwide [53,54].

*Listeria monocytogenes* is a Gram-positive, facultative, intracellular, rod-shaped bacterium incriminated as the causative agent of a severe food borne illness in humans contacted through the fecal-oral route and characterized by localized cutaneous infections [28]. Dairy cows serve as the main reservoir and high levels of the pathogen occurs in animals fed with improperly fermented silage contaminated by growth on manure-fertilized soils. Animal listeriosis is manifested as encephalitis, meningitis, septicemia and abortions. The organism’s ability to withstand heat, freezing and refrigeration temperatures have been a call for concern in the food industries since raw foods, unpasteurized milk, raw vegetables, raw and cooked poultry and refrigerated foods could serve as vehicles of transmission [55,56]. This characteristic is contradictory since it is a non-spore former. Notwithstanding, it can grow over a wide range of environmental conditions (pH and temperature) thus a longer survival duration in the environment [57].

*Yersinia enterocolitica*, a member of the genus *Yersinia* is a Gram-negative cocobacillus belonging to the family of *Enterobacteriaceae*. It is unsporulated, non-capsulated and lives as an intestinal flora in many species of wild and domestic animals, including cattle as well as humans [58]. It causes yersiniosis in both animals and humans manifested as lymphadenitis, acute enterocolitis, nosodum erythema, septicemia, poliartritis and even death [32]. According to Tirzui and colleagues [32], the cases of yersiniosis infections throughout the world are under reported and the prevalence/incidence varies from country to country owing to the availability of materials, laboratories and engagement of specialists. The species *enterocolitica* presents with several serotypes, however, *Y*. *enterocolitica* O:9 demonstrate cross reactivity of its smooth liposaccharide with that of *Brucella*, which masks the diagnosis of brucellosis caused by the latter bacterium [59]. Its indirect route of transmission is mainly oral ingestion of contaminated foods of animal and plant origins (e.g., pork, beef, lamb, unpasteurized milk, raw milk, vegetables, ice creams and seafoods) as well as contaminated water [60,61].

*Enterococcus* species represent a subgroup of the group D fecal *Streptococcus* and as a coccus, they are spherical in shape and occur either singly, in pairs or as short chains [62]. They are Gram-positive, facultative anaerobic, lactic-acid producing bacteria that live as commensal bacteria in the gastrointestinal tract of humans and animals [63]. Members of this subgroup include *Enterococcus faecalis*, *Enterococccus faecium*, *Enterococcus gallinarum* or *Enterococcus* *casseliflavus*, *Enterococcus mundtii* and *Enterococcus avium*, which exhibit significant differences in the incidence of virulence factors, antibiotic resistance genes and distribution in fresh and dry cattle manure [62,64,65,66].

*Mundtii* is the species most commonly reported in cattle manure [67]. Generally, the enterococci are considered avirulent and harmless; however, *E*. *faecalis* and *E*. *faecium* are notable opportunistic pathogens causing nosocomial infections in humans [68]. According to the United States Environmental Protection Agency [69] recommendations, enterococci and *E*. *coli* are the two bacteria regarded as indicator organisms used to determine the sanitary quality of recreational waters across the globe.

*Mycobacterium* species are acid-fast bacteria reported to be found in cattle manure. They can survive in store manure/environment for long duration since they can withstand fluctuations in temperature and pH, dehydration and exposure to sunlight. This predisposition is ascribed to their acid fast characteristic established by the high lipid and wax content of the mycobacterial cell wall. In addition, the cell wall components confer upon these bacteria the propensity to repel or not to absorb water causing them to demonstrate less susceptibility to some chemical disinfectants [70].

*Mycobacterium avium* subspecies *paratuberculosis* or *M*. *paratuberculosis* is the primary species reported in manure and is the causative agent of Johne’s disease (paratuberculosis), a chronic disease of the intestine. It is equally linked to Crohn’s disease (a chronic inflammatory bowel disease) in humans. Following infection of the herd, the pathogen can be shed in feces for a long time prior to the physical manifestations of signs and symptoms associated with the disease; in this way, the condition creates great economic loss (to the producer or dairy industries) noticeable as shortened animal’s lifespan, reduced milk production, reduced carcass value and poor reproductive performance [71]. However, *M*. *bovis* or *M*. *tuberculosis* could be excreted through feces only by a small fraction of tuberculous animals and can be transferred through aerosolized manure emanating from tank agitation and or land application of slurry.

*Bacillus* and *Clostridium* species are spore forming bacteria that are commonly found in cattle manure. They belong to the classes Bacilli and Clostridia respectively, but both belong to the phylum Firmicutes [72]. They have the tendency to form spores and persist in that resistant and dormant state when growth conditions are unfavorable but revert to vegetative cells through germination as growth conditions becomes favorable. As spores, they are insensitive to heat, desiccation and disinfectant; consequently, these bacteria were detected in cattle manure though in reduced numbers after pasteurization at 70 °C for hours in a study conducted by Marañón and colleagues [73].

*Bacillus* species identified so far in manure entailed *B. anthracis*, *B. cereus*, *B. subtilis*, and *B. thuringiensis*, which are Gram-positive rods, aerobic spore formers and are mostly harmless and can persist for over years in the soil. The most peculiar of these species is *B.* *anthracis* that causes anthrax (a life threatening and dreaded disease) especially the pulmonary form through the inhalation of the bacterial spores [74]. The class Clostridia consists of Gram-positive anaerobic spore-forming bacteria that are ubiquitous in the gastrointestinal tract [72] and can be divided into two groups based on their ability to invade and multiply within living tissues. *Clostridium tetani* and *Clostridium botulinum* belong to the first group with little or no ability to invade and multiply in living tissues; however, their pathogenicity is manifested by the production of powerful toxins. The second and larger group constitutes *Clostridium chauvoei*, *Clostridium septicum*, *Clostridium haemolyticum*, *Clostridium sordellii*, *Clostridium perfringens*, *Clostridium difficile*, and *Clostridium spiroforme* that are able to invade and multiply in living tissues or the intestines of the host animal. In addition, they produce less potent toxins in comparison to the former group [36]. Based on the individual species, some are responsible for diseases, including mastitis, blackleg, hemoglobinuria, malignant edema and infant botulism in cattle and humans, respectively [75].

In a nutshell, it is remarkable that infections caused by the above-mentioned pathogens could be prevented/controlled through the implementation of stringent sanitation policies and appropriate hygiene measures by food producers and better personal hygienic practices by individuals.

### 2.2. Factors Affecting Survival Rates of Pathogens in Cattle Manure

The survival rate or duration of persistence of each bacterial pathogen in manure gives an indication of its threat to cause infections and diseases. The survival rate varies from a few days to several months, depending on the particular bacterial species being monitored and its ability to respond to the hostile environment of the cattle manure into which they excreted [23]. All the way through literature, there appear to be controversies in the recorded survival rates or die off rates of microbes. This is because laboratory-based studies executed under controlled conditions generated results that differed from those in field-based seasonal studies that were exposed to external influences from the environment. Actually, in a study conducted by Kudva et al. [76], the findings highlighted that the survival period of pathogenic bacteria was lower in laboratory studies as opposed to observations in field studies which were influenced by fluctuations in ambient temperature. As a consequence, the emphasis has pointed to the fact that laboratory results are difficult to apply in field conditions [77]. Notwithstanding, for the subsequent use of manure either in recycling, land application or disposal, destruction of the aforementioned pathogens is very pivotal [78]. Several authors have reported the multiplicity of factors that affected the inactivation of pathogens in manure and these included temperature, pH, moisture content, nutrient availability/organic content, biological interactions, time and the density of the organisms in manure [23,77,79].

Generally, the survival of bacterial pathogens in manure can be influenced by the characteristics of manure (i.e., liquid manure, slurry manure or solid manure), which depends on the management and on-farm practices which in turn determine the method of treatment as well as the microbial makeup (in terms of diversity and load). More elaborately, some farms use water to flush manure off the cement floor in free stalls into separator pits [80], while others may employ scraping or vacuum systems for collection of the manure off the floor and pile them in heaps or others may mix the manure with waste water from rinsing milking equipment and cleaning installations thus diluting the manure. In addition, the characteristics of manure can also be related to the type of cattle, the diet consumed by the animal, the time of the year as well as the conditions and the duration of storage [73].

Furthermore, enteric pathogenic bacteria can survive for a prolonged period in slurry manure owing to its high moisture level, low solid content and the prevailing alkaline pH since it is a mixture of feces, water and urine [81]. The majorities of bacteria survive most favorably in a pH range of 6–7.5 but live for a shorter period in an acidic pH milieu. Temperature and moisture have been considered to be major factors inactivating bacterial pathogens [82]. Wang et al. [22], in a temperature-controlled study, observed and noted the tremendous overall effect of temperature on the survival of fecal coliforms, *E*. *coli* and fecal *Streptococcus*. However, *E*. *coli* recommended as an indicator of public health monitoring is the most thermo labile amongst these three organisms, thus fecal *Streptococcus* is suggested as a suitable indicator because they demonstrated longer survival periods and have greater thermal stability. Nevertheless, pathogen inactivation can occur in manure/slurry stored in batches at low or moderate temperature over an extended period of time due to the influence of UV radiation from sunlight [83].

Solid manure could be stored in heaps in the animal housing to create an interim storage period to reduce the bacterial pathogen level. This process is called build up during which the anaerobic and coliform bacteria at the middle and bottom of the heap will encounter less unfavorable conditions. Even though the temperature required for pathogen inactivation is not reached in these sections [84], destruction of pathogens may result from other chemical factors such as the metabolic end products (volatile acids) resulting from the metabolic activities of microbes existing within these areas since the heap is unturned [85]. Notwithstanding, at the top where there is aeration, elevated temperatures are achieved to cause destruction of pathogens. However, there is a small risk of pathogen contamination from the cooler and interior areas of the heap. Contrarily, Nicholson et al. [30] observed that the die off rates of pathogens on the surface of the heap was similar to those in the main body and ascribed inactivation to increased temperature and concentration of gaseous ammonia.

On the other hand, in scenarios where solid manure is stockpiled outside, i.e., exposed to the environment and ambient temperature, the survivability of pathogens will be influenced by the seasons or time of the year as the environmental temperature varies and fluctuates from winter (cold) to summer (hot) months and from wet to dry seasons. Owing to the lower temperatures in winter, pathogens have been reported to demonstrate longer survival times as oppose to summer season [86]. Explicitly, some pathogenic organisms, especially *E*. *coli* and *Salmonella* spp., are not heat loving thus will rapidly die off when exposed to high temperatures. Berggren et al. [87] observed that the reduction rate of *E*. *coli* in store manure was very slow during cold climate conditions, i.e., it was dependent on the temperature; the pathogen survived longer at 4 °C than at 15 °C and 25 °C. Additionally, Becker et al. [88] also noted the reduction of bovine-derived *E*. *coli* at temperatures typical of summer in Texas and suggested that manure should be applied on agricultural land during this season to ensure a rapid die off rate. In contrast, *E.* *faecalis* and *L. monocytogenes* can survive for longer periods despite the variability in the temperature conditions [30,78]. In addition, decomposing fungi and non-pathogenic microbes from the soil may invade and create an abode in the pile and consequently, act as predators on pathogens thus affecting their survival.

Furthermore, LeaMaster et al. [89] noted that pathogenic microbes normally constitute a smaller fraction of the entire microbial makeup (in terms of microbial load/level) of manure unlike the non-pathogenic counterparts which display varying metabolic activities within the manure milieu. These pathogens are capable of utilizing and metabolizing readily, available and simple organic compounds as opposed to the non-pathogenic microorganisms that metabolize complex organic molecules. In the presence of limited available nutrients and organic molecules, the pathogenic bacteria populations are faced with stiff competition from the non-pathogens; consequently, these pathogens are starved out hence negatively affecting their growth and survival rate.

## 3. The Methods Involved in the Control of Pathogenic Microbes in Animal Manure

The available methods used for the treatment and or management of manure can cause reduction in the level of pathogens which otherwise are capable of causing food and waterborne diseases in humans. Zoonoses represent human diseases caused by animal pathogens [90]. It appears that proper animal care and adequate management of manure can minimize the introduction of these pathogens into food chains, environment and their ultimate transmission to humans [91].

In addition, Spiehs and Goyal [5] affirmed that vaccinating the animals, providing adequate access points for water and food consumption, temperature and ventilation control systems and appropriate space allowance could be an easy first step to reduce the level of the pathogens. They further mentioned that stringent on-farm sanitation and biosecurity measures as well as modification of animal diets by adding supplements and or selection of a particular diet ration could equally go a long way to reduce the number of pathogens excreted in manure that could end up in the environment. On the other hand, Harrison et al. [92] reported that environmental hazards could be minimized by collecting and storing manure, minimizing its runoff from animal housing and storage facilities and finally to prevent direct access of animals to surface water.

Clearly, it has been well documented that the control and destruction of microorganisms have a substantial role within the public health context and can be achieved via biological, physical and chemical methods or a combination of these. Albeit, the overall objective of these treatment methods is to yield the removal of gross solids and hamper the polluting effect attributed to the composition of manure [40]. Thus far, the varieties of methods that have been utilized in the proper management of cattle manure are faced with challenges, although, they equally present with beneficial roles [18,93]. Owing to the differences in the rate of inactivation of the pathogens found in manure by the different control methods; it is somewhat reasonable to employ multiple control methods in a bid to yield significant reductions in the levels of the different pathogens in manure anticipated for further use. This means, the digestate of an aerobically or anaerobically treated manure can be air-dried on sand or concrete slabs for the complete decomposition of partially digested material by aerobic microorganisms guaranteeing a further reduction in the pathogen level.

### 3.1. Chemical Methods

(a). Lime Substance

Lime stabilization of slurry and solid cattle manure has been viewed as a means of treatment whereby lime products such as calcium oxide (CaO) or calcium hydroxide [Ca(OH)] are used as chemical compounds to destroy pathogens present in the manure. In this process, an adequate portion of lime is mixed homogeneously with cattle manure to elevate the pH of manure to 12 for ≥2 h of contact [17]. Inactivation/destruction of microbial pathogens by lime products occurs as a result of an increase in pH added to the inhibitory effect exerted by ammonia released at such a high pH of above 10 [18]. However, Heinonen-Tanski et al. [94] reported that two days were to some extent satisfactory for the destruction of intestinal microorganisms to a level below detection limit.

Spiehs and Goyal [5] noted the advantages of lime treatment to reduce odor and pathogens in cattle manure prior to land application and also its cost-effectiveness, easy disposal of the treated manure as well as the reduction of soil acidity. Its shortcoming is attributed to the large quantity of wastes that results after treatment and has to be disposed off. The increased weight of the wastes is due to the addition of the chemicals (which are materials not generated on the farm); this calls for more expenses (i.e., increase in transportation cost). Furthermore, careful planning is necessary to determine the calculated amount of wastes to be treated and to what quantity of the particular chemical compounds has to be made available. In addition, specialized instruments/apparatus may be relevant in circumstances where vigorous and prolonged agitation of the mixture is crucial. At the end of the process in circumstances where other chemicals are employed for sanitization, the end product required to be disposed of may be endowed with the potential to cause greater environmental risk than the initial microbial pathogen themselves [95]. In addition, the suspended solids in the slurry could cause the outcome of the treatment to be unsatisfactory as it might interfere with the biocidal activity of some chemical disinfectants [96].

(b). Hydrogen Peroxide

It is an oxidizing agent and an aqueous solution that varies in percentage concentrations from 3% (where it is being used as a disinfectant) to 98%. Its mode of action as an oxidizing agent relies on chemical processes that results in the destruction of cellular structures of pathogenic bacteria either completely or partially by raising the pH value above 12. At such given elevated pH, a broad spectrum of microbial activity is limited [97]. Bilotta and Kunz [97] emphasized the need of pretreatment of the waste to remove suspended solids, which may influence the efficiency of the disinfection process. Clearly, the increase in total solids in store manure hampers solid degradation via natural processes undertaken by indigenous microorganisms.

Besides the disinfection effect, hydrogen peroxide aids in odor and gas emission controls as well as causes clarification of the final effluent [98]. Unfortunately, it can be toxic and costly.

### 3.2. Physical Methods

These methods include heating (pasteurization and burning), air-drying and irradiation (gamma, alpha and UV radiation).

(a). Pasteurization

It is a thermal process during which manure is heated above a certain predetermined temperature for a minimum period of time. The pasteurization process should be conducted by a well trained personnel and manure is heated by heat exchangers or by steam injection [99]. It is noted that it eliminates zoonotic pathogens without spores, whereas the bacteria harboring spores are only reduced. The pasteurization method can be used as a pretreatment method prior to application of other known methods of treatment. However, it can be conducted before or after anaerobic digestion in a batch or continuous mode; the batch operation is often recommended as it can be easily controlled with respect to temperature and time [73]. Pre-pasteurization is acceptable since post pasteurization has been reported to be vulnerable to recontamination. Notwithstanding, the contact time for the process depends on the subsequent anaerobic digestion; a pretreatment of animal waste is done at 70 °C for 60 min or 30 min before mesophilic and thermophilic digestion, respectively [100]. In this regard, Marañón et al. [73] pre pasteurized cattle manure for 2 h, followed by mesophilic anaerobic digestion in an up flow anaerobic sludge blanket which resulted in the removal of pathogenic bacteria without spores including *Yersinia*, *Pseudomonas*, *Enterococcus* and coliforms.

For local information gathered, inhabitants in rural settings in South Africa equally burn relatively dry cattle manure for cooking. This process will eventually cause a drastic reduction in the level of intestinal pathogens present in the manure but would destroy all the nitrogen and organic matter available, and, consequently, will affect the quality of fertilizer that could be used to increase the humus content of the soil [1]. Furthermore, it is a simple and fast technique, however, its main drawback is that spores of pathogenic microorganisms are not removed but only reduced [73,101].

(b). Air-Drying

Air-drying of animal manure involves drying the manure material on sandy bed or on paved or unpaved basins during which the organic material contained therein is biologically decomposed, ammonia is produced and the material will become desiccated [99]. The effectiveness of this method depends on the duration/period of drying, which is dependent on the local climate implying a shorter length of time in a warm, dry weather or summer season as oppose to a longer time period in a cold, wet weather or winter season. However, the minimum time lapse for drying should be three months during which the ambient average daily temperature has to be above 0 °C. Manure treated by drying should be pretreated by aerobic or anaerobic digestion to partially decompose the organic matter that could present with odor thereby causing vector attraction that may serve as a plausible route of pathogen transmission [17]. This method is predisposed to destruction of pathogens by natural means of predation and sunlight irradiation.

(c). Ultraviolet Irradiation

Comprehensively, the sun is the major source of ultraviolet radiation. UV radiation is observed as a promising technology engaged in the disinfection of cattle manure for bacterial control. It could be divided into UV C, UV B and UV A depending on the wavelengths and energy intensity. However, due to the high energy level of UV C, it is absorbed by the ozone layer of the earth stratosphere. UV A (315 to 400 nm) and UV B (280 to 315 nm) radiations are employed in the disinfection process, however, Willey et al. [93] noted that UV radiation around 265 nm is quite lethal. In a study conducted by Oni and colleagues [102], a 5log reduction of *Salmonella* cells was observed when exposed to UV-A (365 nm) in a control medium as opposed to a 1.5log reduction in a manure dust matrix.

In addition, UV radiation is an efficient disinfectant however; its efficiency is dependent on the type of microbes, the intensity of the UV light source, the contact time, i.e., the duration of exposure to the radiation as well as the quantity of suspended solid materials in the manure samples. Bilotta and Daniels [103] in their findings noted that *E*. *coli* cells in sewage was a 100,000 times (99.999% or 5log) reduced after exposure to UV radiation for 60 s at a dosage of 200 mJ/cm^2^. The process destroys the DNA and RNA of the microbial cell by causing thymine-thymine dimerization [93], resulting in the formation of distorted proteins or even death of the cells. The significance of UV irradiation is that it disinfects the manure without the use of any chemicals consequently; no disinfection by-products are generated so it does not alter the physicochemical and nutritional properties of the manure after treatment [5]. In fact, only the microbial cell that had absorbed the energy at that specified wavelength is destroyed [103]. The drawbacks of this method include; no residual disinfection as destruction of microbes takes place only within the treatment unit and not after treatment to remove the survived cells or the new introduction of pathogens after treatment, as well as different skin infections and eye problems may occur.

However, gamma, alpha and UV irradiation of manure are only acceptable theoretical physical methods of pathogen control. They are very cost-intensive and cannot be economically appropriate for use by small-scale farmers [5].

### 3.3. Biological Methods

Manure is regarded as a vital source of nutrients (nitrogen, phosphorus, potassium, etc.) and micronutrients (zinc, copper, iron, etc.) required for growth by crops and can influence soil productivity [2]. Overall, the physical, chemical and microbial characteristics of cattle manure guarantee an avenue for biological treatment as it makes use of naturally occurring microbes to break down dissolved and particulate biodegradable materials in manure in the presence or absence of oxygen. These biological processes include anaerobic lagoons, aerobic and anaerobic digestion and composting [104].

(a). Anaerobic Storage in Lagoons and Tanks

Farm-based activities usually require storage facilities to safely collect the animal wastes generated. Traditionally, lagoons are used to collect animal wastes in a liquid, semisolid or solid form for storage and sanitation purposes [105].

The storage facility is placed distances away from surface water and is normally placed on high, well-drained ground with concrete floor and covered over with a roof [3]. Utilizing storage facilities as a means of pathogen reduction recommends that fresh manure should not be added after a certain period of time, allowing the stored manure to undergo the process of aging. However, no significant reduction in pathogen will be achieved if fresh wastes are continuously added to an aging store of manure. This can be shown in the findings of Hutchison and coworkers [4] who recorded no significant differences in the pathogen levels of groups of stored manure samples of different ages. They attributed this contradiction to the fact that these wastes were not managed in waste stores as batch operations. Accordingly, more than one storage tank or lagoon are needed; such that one is being left for treatment (aging) whiles the other continues receiving the manure because all the manure needs to be in the lagoon for a specified duration for treatment [106].

Generally, to record a significant reduction in pathogen load during storage, the holding times are longer under colder conditions than warmer conditions insinuating treating or storing manure in a lagoon/tank at a temperature of ≤5 °C requires a period of at least six months or at a temperature of >5 °C warrants a period of at least four months. In other words, the declining rate of pathogens in store manure depends on storage conditions and management methods [30], but also on the particular characteristics of each organism. This may suggest that the decay rates of *E. coli*, *Salmonella*, *Campylobacter* and *Listeria* species differ from each other and even at different temperatures. This is in accordance with the findings of Nicholson and co-workers [30] who demonstrated that the former species survived for a month while the latter survived for up to six months in dairy slurry stored at <20 °C. Furthermore, variation in the reduced rate of a single organism may correlate with variation in storage temperature of the stored manure. This congruents with the findings of Berggren et al. [87] which demonstrated that *E*. *coli* O157 was greatly reduced in the order 25 °C > 15 °C > 4 °C in an infected cattle manure stored at three different temperatures.

In addition, management encompasses the following processes; collection of manure, storage, mixing, pumping, spreading and treatment methods [107]. These processes vary from one farm to another and are influenced by economic feasibility. Clearly, manure on the dairy farms could be collected by vacuum, flush and scraping systems resulting in slurry, liquid and solid manure respectively, with different characteristics. Furthermore, the bacterial level and composition vary across these three categories of manure owing to the fact that some farms mix manure with waste water generated from rinsing milking equipment and from animal free stalls. Subsequently, when stored at a particular temperature, there is the possibility of varying reduction rates amongst the microbes thus affecting the level of microbes contained therein. Furthermore, employing treatment as a mode of management, it is evident that diverse reactions are inevitable since different microbes respond in varying ways to the different treatment methods due to their inherent characteristics as well as the operational and external factors.

The advantages of storing manure in lagoons consist of the following; It makes manure to be conveniently and easily handled during land application, it provides long-term storage at a relatively low cost, it minimizes odor pollution due to stabilization of the organic matter and it causes reduction of nitrogen concentration. The shortcomings include; it requires relatively large area of lands to include the structure, it takes quite a longer period of time for organic matter to be stabilized and sanitized, can cause deleterious effects to environment and humans through gaseous release and lagoon overflow (due to cracks in improperly designed and build structures, persistent heavy rain and strong winds) [3,5,105,106].

(b). Composting

This could be conducted under two conditions; either in the presence of oxygen (aerobically) or without oxygen (anaerobically). Notwithstanding, aerobic composting is preferred owing to a greater amount of heat released as opposed to the anaerobic counterpart. Hence, aerobic composting is an efficient biological process during which indigenous aerobic microorganisms decompose organic matter contained in manure under controlled temperature, oxygen and moisture concentration to release carbon dioxide, water vapor, and heat in addition to an agronomically enhanced soil conditioner (compost) [5]. An Appropriate C/N ratio of 20 to 40:1 and 40% to 65% water content is highlighted as prerequisites for a good composting medium [108]. Basically, there are three common methods of composting viz. windrow, aerated static pile and within-vessel methods; all of which require bulking materials or carbon amendments e.g., wood chips, peat and straw in a bid to create a nutrient-rich environment required to optimize microbial growth or drive the metabolic activities of thermophilic microorganisms.

The quantity of heat produced during the process depends largely on the type and age of bulking agent and also its surface area since these determine the availability of carbon to the microorganisms [109,110]. Explicitly, carbon from cellulose contained in the straw is more readily available to microbes than carbon from lignin stored within wood chips; increasing the surface area of the same carbon amendment also results in its greater availability to microorganisms [111]. As a consequence, more heat is generated from metabolic break down by the microbes occurring in a compost heap constituted of straw than that of wood chips. Additionally, an older feedstock that has undergone the decomposition process offers a readily available carbon source for microbial activities thus generate more heat. Nevertheless, carbon amendments are essential during the composting process to reduce ammonia volatilization [112].

More elaborately, in windrow composting, manure is mixed with the bulking agent and stacked into long piles or windrows of height, 5 to 9 feet and width, 9 to 20 feet. These piles are often turned or mixed to provide oxygen to the microbes engaged in the decomposition process [108]. In aerated static pile composting, manure is either mixed with the bulking agent and positioned on a fixed forced aeration system underneath or on top of a layer of manure is placed perforated pipes and both are covered with a layer of bulking material. As a final point, a layer of cured compost is used to cover the entire pile. This coverage insulates the pile, absorbs water, control odor and prevents the composting materials from vectors. Furthermore, within-vessel composting occurs in a reactor vessel making it possible for the operating conditions to be controlled [99]. The difference between these methods lies in the mode of oxygen supply to the composting material coupled with the duration of composting.

It is worth mentioning that after the initial five days of composting at the elevated temperature, the end product (compost) still harbors volatile solids that causes odor. As a consequence, the complete breakdown of these volatile solid is achieved in a within-vessel, static aerated pile and windrow over a period of 14 to 21 days, 21 or more days and 30 or more days, respectively. Overall, the process ends up reducing the weight of manure by 50%–80%, thereby making the cost of transportation of treated manure or compost less expensive [104].

On the whole, the key features of all the methods include, primarily, oxygen is supplied into the windrow, static aerated pile and within-vessel composting manure. However, these different methods implement different modes of supplying oxygen to the manure during composting. Seemingly, windrow and some types of within-vessel method make use of mixing or turning over method that aerates, reduces moisture content, increases porosity of the composting material as well as breakdown compacted material [8,108]. Alternatively, perforated pipes are incorporated within an aerated static pile to allow air flow [108]. In addition, the temperature of the animal manure is raised to 40 °C or above and is maintained at the said temperature or higher for five days [99]. In addition, the temperature exceeds 55 °C for 4 h during the five-day retention time. Heat is released from the microbial metabolic processes involved in the decomposition and it is pivotal to the destruction/inactivation of the pathogens present [109].

On the other hand, proteins are degraded, resulting in increased levels of pH and ammonia that equally affect the survival rate of pathogens [18]. In addition, Erickson et al. [113] also indicated that other factors such as non-lethal temperature, light, desiccation, volatile acids, competition for nutrients and other antimicrobials (bacteriocins) affect pathogen survival in manure-based compost heap. Desiccation occurs as a consequence of the high lethal temperature generated in the compost heap which removes moisture from it causing the surface to become drier. However, the efficacy of the method to destroy pathogens relies on the degree of temperature and the period of time the pathogens are being exposed to the heat [19]. Furthermore, Larney and colleagues [114] reported that more than 99.99% of total coliform and *E*. *coli* were inactivated within seven days in cattle composted by windrows technique thus indicating over 4log reduction of bacterial cells. In addition, Sunar et al. [115] demonstrated 4log reduction of *Salmonella* spp. in the composting material after five days of composting and 7log reduction after 20 days as the temperature increased to 64 °C. This indicated 99.99% to 100% reduction of these bacterial cells during the 20 days of composting.

It is certain that the process of aerobic composting occurs via four successive stages conducted by different groups of microorganisms that predominate at each stage guided by the prevailing temperature at that stage [84]. These include mesophilic, thermophilic, cooling (these three stages constitute the biooxidative phase) and curing/maturation stages where mesophilic and thermophilic bacteria as well as fungal and actinomycetes microbes are involved [116]. Martin [8] in his findings advised that irrespective of the method used in composting, the compost should be further composted for several weeks before land application through a process known as maturation or curing. The curing stage is very beneficial as it stabilizes the finished compost with remaining nutrients into microbial metabolic products and also ensures safety by allowing the inactivation of the residual pathogens that may be present. Nevertheless, compost maturity is very much dependent on the activities of the indigenous microbes during the composting process [108]. After this time, compost can be purchased as a high-value residential, potting medium, bedding and serves as an organic farming market added to the fact that compost teas can be produced by packaging small quantities of mature compost in perforated bags and soaking them in water to create a compost tea solution suitable for soil application [104].

In contrast to the aerobic counterpart, anaerobic composting is a breakdown process through fermentation by anaerobes, of nitrogen-rich organic materials in the absence of or in the presence of limited oxygen resulting in the generation of methane, ammonia and sulfide gases (e.g., Methane and hydrogen sulfide) that might create severe odor nuisance. Herein, the pile composted is not turned throughout the entire process thus it is less labor intensive. As a result, heat is not generated that will help in the sanitization of the materials by destroying weed seeds and pathogens. Moreover, this process is slower and retains the weed seeds and pathogens will have to disappear gradually over an extended period of time owing to unfavorable environmental conditions and biological antagonism [117]. Due to the slow nature of the process, volume reduction of the piled materials is less and more humus is generated as opposed to the aerobic counterpart. In addition, the compost produced is dark, wet and slimy, but endowed with nutrients relevant for soil enrichment and plant growth, therefore it is aerated for six months to one year for further maturation and elimination of pathogens and weeds [118].

Overall, the advantages presented by aerobic composting entails; aids in minimizing the quantity of organic materials to be disposed into landfills, enables easy handling for application of manure into the soil, increases the bioavailability of soluble nutrients for plants uptake by converting them into more stable organic forms [84], well managed compost pile generates lethal temperature responsible for the substantial reduction of pathogens [119], produces an end product (compost) used as a soil amendment which; increases soil quality, structure, fertility and microbial activity [120], improves on the water absorption and retention property, promotes granulation and increase in cation (Ca^2+^, Mg^2+^, and K^+^ produced during decomposition) exchange capacity and diminishes the use of conventional chemical fertilizer [121,122].

Limitations of composting documented include; leaching of liquid from the compost pile to underground springs and surrounding surface waters, odor pollution, serve as attraction sites for vectors, creates dust, which can cause allergic reactions when inhaled by humans, loss of nutrients such as nitrogen, causes ammonia and carbon dioxide (a green house gas) emission into the environment, compost materials might be washed by run-off into water bodies, the lack of available labor, time, equipment and land for storage and operations [116].

Vermicomposting on the other hand, is a biooxidation process involving the combined action of microorganisms (bacteria and fungi) and earthworms to stabilize organic matter and extensively augment the physical and biochemical properties of the original material. Unlike composting, it is a mesophilic process and as a consequence, the thermal stabilization of organic matter to inactivate or destroy pathogens is absent [123]. Nevertheless, Aira et al. [124] documented that the reduction of the pathogen level via vermicomposting is dependent on the species of earthworm employed and/or the pathogen measured. Therefore, depending on the specific microorganism, its transit through the gut of a specific earthworm may cause its elimination. More elaborately, predation is one of the biotic interactions occurring between the bacteria and earthworm in a vermireactor [123]. In addition, the gut of the earthworm has a selective effect on specific microorganisms which respond differently during their transit with the organic waste through the gut in that some may be activated, digested in the intestinal tract (this causes a reduction in number) while others remain unaffected [125].

Moreover, Monroy and colleagues [125] and Edwards [126] noted the reduction of total fecal coliforms in a vermicomposting process by 98% and attributed the elimination of the coliforms to the digestive potential of the earthworms. These included the fine grinding of cells and the high number of available enzymes responsible for the degradation of bacterial cell wall. These digestive abilities of the earthworm gut vary from species to species and thus play a major role in the reduction or elimination of the pathogenic microbes. This indicates that the bacteria, including *E*. *coli*, *S*. *enteritidis*, *C. perfringens*, total coliforms will respond differently during gut transit in these species of earthworms, *Eisenia fetida*, *Eisenia andrei*, *Lumbricus rubellus*, etc. thus affecting their reduction level. In addition, Suthar [127] remarked that vermicomposting generates an end product (vermicompost) of a better quality in terms of nutrient availability than the conventional composting technology.

In the face of these obvious differences, Tognetti et al. [128] highlighted that combining composting and vermicomposting will in a way achieve stabilization of the substrates by sanitizing the wastes and eliminating the toxic compounds via composting while the nutrient availability and particle sizes are being increased and reduced respectively, through the latter process. Interestingly, Ndegwa and Thompson [129] commended that inoculation of earthworms into a substrate produced from the thermophilic stage of composting could result in shortening of the process as well as cause a reduction in the expenses. This is in line with the work of Lazcano and coworkers [130] who demonstrated that the combined treatment of composting and vermicomposting was the most effective in stabilizing cattle manure unlike composting and vermicomposting processes performed in isolation or as standalone processes.

In vermicomposting, microorganisms are responsible for the biochemical decomposition of the organic matter, whereas the earthworms display crucial functions that affects the overall decomposition process by either directly preying on microorganisms, thereby hampering the microbial decomposer activity and also increase the surface area for microbial attack [131]. In addition, the earthworms act as mechanical blenders and are the crucial drivers of the process functioning as turning, fragmentation and aerating agents during vermicomposting [130]. It is obvious that the maturity/stability of the end product known as vermicompost is dependent on the species of earthworm available in the process since they have the tendency to stimulate or depress or modify the microbial community structure and activity, thus they would have different impacts on the decomposition process that will virtually determine the properties of the vermicompost [123,132].

According to Singh et al. [132], vermicomposting is beneficial in that; it is odorless, pathogen and pollution free, cost effective and results in a valuable product with nutrients essential for plant growth in more soluble and available forms, low green house gas emissions as well as reduce the volume of the wastes thus making its application easier. In addition, it is a rapid process that removes and accumulates heavy metals within the bodies of the worms added to the breakdown of complex chemicals to nontoxic forms making certain its safety [132].

(c). Aerobic Digestion/Aeration

USEPA [99] described aerobic digestion, as a process in which the animal manure is agitated with air or oxygen for a specific solids retention time at a specified temperature and can operate as a batch or continuous system. Specifications for the required mean cell residence time include 40 days at a temperature of 20 °C and 60 days at 15 °C.

(d). Anaerobic Digestion

Babaee et al. [133] described cattle manure as an organic and an inorganic rich substrate with high levels of cellulose and hemi-cellulose that also present with an appropriate C/N ratio of 20 to 30:1 which is suitable as nutrients for anaerobic microbes that are involved in anaerobic decomposition. Anaerobic digestion of cattle manure represents one of the most environmentally friendly treatment methods employed to control the population of pathogens to a level acceptable for public health safety [16]. In details, it is associated with minimal or negligible effects on the environment (i.e., air, water, land, humans and microbial components). More elaborately, methane and carbon dioxide, the green house gases, are the main gases produced during the anaerobic degradation process within the confinement of the digester. These gases are implicated in climate change when released into the atmosphere, but in this process, they are restrained within the airtight chamber, thus, it is a potent tool to mitigate climate change and global warming [134,135]. Seemingly, the process reduces odor because the anaerobic breakdown of substrates under uncontrolled environmental conditions is usually incomplete, resulting in the formation of malodorous end products/compounds that have the potential to pollute the air [136].

According to Arthur et al. [137], anaerobic digestion of such waste generates biogas, which is lighter in terms of carbon chain length and burns with the release of less amount of carbon dioxide into the atmosphere as well as leaves no soot or particulate matter. In addition, it provides energy and can be further harnessed into electricity. In addition, water sources, both surface and underground are greatly protected from pollution due to treatment of the raw manure within a confinement where it becomes stabilized and sanitized [137]. Moreover, the stabilized material (digested slurry) with high nutritive value can be applied to soil as a conditioner/fertilizer to protect the land from erosion and depletion [138].

Furthermore, the process of anaerobic digestion of cattle manure is defined as a natural biological process occurring in an airtight chamber during which complex organic materials contained therein are being degraded into simpler molecules through four stages (hydrolysis, acidogenesis, acetogenesis and methanogenesis), by the combined activities of four groups of metabolically linked microbes in an oxygen free environment [16]. Anaerobic digestion can occur both at mesophilic (25 to 37 °C) and thermophilic temperature (55 to 65 °C) ranges either as a batch or a continuous system. Amongst the benefits presented by anaerobic digestion, it has also been associated with a reduction of pathogen occurring in cattle manure. Inactivation of these zoonotic pathogens does occur under both temperature conditions, however; the degree of reduction in mesophilic temperature is lower compared to thermophilic temperatures [139].

Although anaerobic digestion at thermophilic temperatures achieves a greater elimination of pathogens; it, however, faces a lot of challenges regarding system performance, i.e., instability resulting from the characteristics of manure. Garcia et al. [140] noted that a higher ratio of free ammonia to total ammonium ion resulting from the anaerobic decomposition of cattle manure posed instability in digester performance. Therefore, anaerobic digestion of cattle manure (contains nitrogen and ammonia compounds) is often performed at mesophilic temperatures thus ensuring process stability and also less quantity of energy being used. However, temperature is not the only factor responsible for pathogen inactivation; pH and the concentration of free ammonia also contribute to the inactivation effect. Seemingly, it can be concluded that the factors affecting anaerobic digestion of cattle manure arise from its characteristics [136] and these factors have been clearly presented elsewhere [16,139,141]. As a consequence, a continuous control strategy to monitor the anaerobic digestion process within the digester is inevitable in order to circumvent problems arising from the operations [2].

Several studies have investigated the survival rates of zoonotic pathogens harbored in cattle manure by employing different methods of treatment, operational and environmental conditions, thus variations existed in the obtained results [109,142]. In the findings of Harrison and Saunders [142], it was reported that pathogenic bacteria were reduced by 90% to 95% through anaerobic digestion at a mesophilic temperature. Similarly, Harrison et al. [92] demonstrated a 2.5log reduction of *E. coli* in manure treated by anaerobic digestion. In addition, Côte et al. [143] used sequenced batch reactors to anaerobically treat manure and recorded a reduction of 99.67% to 100% of indigenous *E. coli* population and achieved undetectable levels of indigenous *Salmonella* strains, and protozoa (*Cryptosporidium* and *Giardia*).

Most recently through mesophilic anaerobic digestion in a balloon type digester, Manyi-Loh et al. [144] documented a 1log reduction of *E. coli* and *Campylobacter* spp. (i.e., 90% decay rate) as opposed to a 2log reduction of *Salmonella* spp. that occurred between day 9 and 14 but a similar 1log reduction of these cells during the rest of the process indicating a 90%–99% kill rate. However, these contradictory results are expected and the plausible factors responsible for this variation could be ascribed to differences in the chemical composition of the wastes, the retention time of the process, environmental factors (temperature), digester operating conditions (batch or continuous mode) in addition to the type of digester being used [101,145]. Furthermore, the physicochemical characteristics of the animal waste are dependent on the weather conditions and soil properties that determine the feed (vegetation) properties of these animals which might equally vary from one geographical location to the other as well as the overall microbial species contained therein also vary with farm practices.

Due to the negative value attached to liquid manure, it can therefore be concluded that anaerobic digestion of cattle manure (liquid and solid) is a relevant step in the sanitization of cattle manure before application on land. This is because the problem of odor has been remedied and the potential of polluting the air, water and land has been drastically hampered as a result of the reduction of zoonotic pathogens that pose health threats both to animals and humans. Furthermore, the organic content of the manure has been stabilized through decomposition by the anaerobic microbes releasing nutrients that are vital for soil improvement and plant growth.

Nevertheless, the process of anaerobic digestion presents with benefits/advantages that include; it helps to reduce global warming by preventing the release of the green house gases into the air which otherwise would have been emitted from stored and land applied manure added to the fact that it uses biomass, a non-fossil material thus reduces the emission of carbon dioxide from fossil carbon origin, it generates biogas (a renewable energy source) which can be used for cooking, direct heating or further harnessed to generate electricity, it produces a biofertilizer (liquid or solid form) that can be used for soil amendment thereby reduces the utilization of chemically synthesized fertilizers that are potentially hazardous to humans and environment alike [139], it increases the bioavailability of essential plant nutrients and it helps in pathogen and odor control [141]. However, the following disadvantages are inevitable such as; it is not cost effective since it requires sophisticated equipment for the monitoring and controlling of the system , it needs the expertise of a well trained personnel or a specialist, it entails a complicated process that needs to be monitored and controlled by a specialist, it does not degrade heavy metals e.g., chromium, cadmium, mercury and lead that might originate from animal feed additives and are toxic to plants, animals and humans [146] and finally, it does not destroy spore forming bacteria [78,147].

More elaborately, with the exceptions of prions and spore-forming bacteria, for example *Clostridium* and *Bacillus*, microbial decontamination in anaerobic digested manure aids in minimizing the pollution potential of the treated manure unlike the raw manure which harbors huge quantities of pathogenic bacteria [27]. In general, the level of microbial sanitization is often considered as a vital parameter for the ecological evaluation of the efficiency of anaerobic digestion of organic wastes described as the number of indicator bacteria notably *Enterococcus* and *E. coli* [148]. In a nutshell, the significance of reducing pathogens in manure treatment is not questionable, but to sustain human health and environmental quality [97]. Generally, methods incorporated in the control of pathogenic microorganisms in manure are equally aimed at treating the manure. Notwithstanding, for more efficient pathogen inactivation anaerobic digestion can be combined with other treatment methods (e.g., aerobic digestion, composting, thermal treatment). However, the primary goal of any treatment method is dependent on the needs of a specific farm.

#### Influence of Compost/Digestate on Pathogenic Load upon Soil Application

Digestate is the by-product either in a liquid or semi solid form emanating from the anaerobic decomposition of organic wastes by anaerobic microbes under controlled conditions (biogas plant) whilst compost is the remaining solid materials after the aerobic breakdown of organic wastes by aerobic bacteria and fungi under controlled conditions [132,149]. These final end products both contain macro and microelements essential for improving soil fertility, soil microorganisms and plant growth; however, according to Tambone et al. [150], the digestate contains a higher concentration of macronutrients such as nitrogen, phosphorus and potassium unlike compost. The stability/maturity of these final products is very imperative and is reliant on the chemical and microbial changes that cattle manure undergoes during both biological processes which in turn determines their safe use as soil amendments [146].

However, there exist controversies in data on the effects of application of digestate and compost as soil amendments (i.e., whether and to what extent are the effects) on soil microbial biomass, community structure and activity. These discrepancies in the findings were ascribed to, among others; differences in the experimental design, soil properties, the type of land used, the dose and frequency of application of the compost/digestate, time of the year, the parameters selected for analysis as well as the duration of the experiments [123].

Once applied into the soil, the pathogen load of the final product is further reduced as some of the pathogens in the broadcasted compost/digestate will be exposed to the ultraviolet radiation from sunlight and become inactivated [30]. The Supply of additional organic carbon source, micro and macronutrients in the soil environment causes changes in the diversity, size and makeup of the microbial community [123,151]. In circumstances where the application of these products is done on soil with healthy, intact microbial community, the fate of enteropathogens introduced into the soil will be influenced by abiotic (soil moisture, structure and pH, available nutrients and supply of oxygen) and biotic interactions (antagonism, predation and competition for food and occupation of niche space). More elaborately, the supply of these nutrients will cause an increase in microbial activities, thereby causing the enteropathogens to face stiff competition and will be outcompeted regarding niche space and food as the native soil microorganisms are better adapted to compete more effectively [152].

In addition, soil type has been reported to have an impact on the survival of enteropathogens in the soil milieu. There exists different kind of soils according to their mineral fraction ranging from sand, silt and clay, whose formation is dependent on the parent material, climate, time and management practices [153]. Each soil type is unique in respect to the water, nutrient and habitat availability, thereby emphasizing that the clay soils unlike others, have the potential to sustain pathogens for a long time owing to its endowed characteristics of fine texture, nutrient absorption and microspore availability [152].

On the other hand, the soil as an ecosystem consists of diverse and abundant microorganisms that are sustained therein by its available water, pH, nutrients and oxygen supply. Consequently, they play critical roles in many biological processes necessary to maintain the fertility and quality of the soil [123]. However, when the soil environment is compromised either via sterilization or excessive use of chemical fertilizers; the enteropathogens introduced by way of amending the soil with inadequately managed compost/digestate will survive for a period of time depending on the specific bacteria since they will be nourished by the inherent soil properties (nutrients, water and oxygen) and the microbial community diversity must have been reduced [138]. Therefore, combating the likelihood of antagonism, competition and predation in the environment. Apparently, the pathogenic load of the soil amended will depend on the initial bacterial concentration present in the compost and digestate, respectively, as final products and also whether the soil environment is compromised or not.

## 4. Recommendations Based on Literature Findings for Better Management of Manure

Livestock farming generates huge quantities of animal manure, which must be properly handled, managed and treated. An age-old tradition of direct application of fresh/raw animal manure on land should be prohibited, thus necessitating the use of proper collection and storage facilities before treatment. Therefore, management and treatment of these wastes should be given great attention by producers, determining which method or combinations of methods are economically feasible for their operations and that will result in the protection of land, air and water [107]. In addition, each method should be evaluated on a case-by-case basis because livestock operations vary from one farm to the next, which in turn might influence the characteristics of wastes generated [5,136]. In addition, since copious amount of animal wastes is being produced, the use of storage devices is plausible. The storage systems of manure should be batch operated, enabling the reduction of pathogenic microbes contained therein. These storage facilities should be well covered to prevent runoff and leaching from stockpiled manure into the environment [4,5]. Owing to the high organic, nutrient and microbial contents of manure, its presence in the environment guarantees devastating outcomes. Furthermore, the type of food and the feeding mechanism should be looked into because some diets influence the microbial composition of the gastrointestinal tracts thus the type of microorganisms that might be shed in the feces of these farm animals will eventually end up in the environment [5,39]. Moreover, public campaigns to sensitize the masses, more especially farm owners on the risks associated with handling raw manure and the necessity of proper management and treatment are imperative. Lack of knowledge is sometimes the cause of the many challenges people might face presently or even in the future. Lastly, further research should be conducted to improve on the existing management practices as well as to uncover new, low cost operations that would minimize pathogen transfer from manure which are subject of intensive investigation in our group.

## 5. Conclusions

From the chronicles of the diversified treatment options presented herein, it is somewhat obvious that the pathogenic bacteria population of public health and environmental significance needs to be controlled by a combination of two or more methods depending on the feasibility and economic viability of the animal farm. In this light, each farm has a right of choice as to what method to taking into consideration the number of animals, quantities of manure generated, size of the storage facility as well as the financial potential of sustaining a particular method of control. Nevertheless, we can deduce from literature that anaerobic digestion in biodigester could be the best method for the control of bacterial pathogens in animal manure as it presents with several benefits that stand to boost the socioeconomic status of the people and the environment. Notwithstanding, it should be preceded or followed by another treatment method.

## Figures and Tables

**Table 1:** Threshold levels of pathogens in compost obtained from different countries.

Country	Pathogen	Threshold Level	Reference
USA Class A biosolid Class B biosolid	*Salmonella* or Fecal coliform Fecal coliform	<3 MPN/4 g DM <1000 MPN/g DM <2 × 10^6^ MPN/g DM	United States Environmental and Protection Agency (USEPA) [12]
France NFU 44-051	*Escherichia coli* *Enterococcus* *Salmonella*	10^2^ per g (wet weight) 10^4^ per g (wet weight) 0 per 25 g (wet weight)	Association Française de Normalization (AFNOR) [13]
Canada	Fecal coliform *Salmonella*	<1000 MPN/g DM 0 with <3 MPN/4 g dried weight	Canadian Council of Ministers of the Environment (CCME) [14]
Switzerland	Enterobacteria Helminth ova	<100/g DM 0	Lepeuple et al. [11]
Italy	*Salmonella*	<100 MPN/g DM	
United Kingdom	*Escherichia coli* *Salmonella*	<1000 CFU/g fresh mass 0 per 25 g fresh mass	Wastes and Resources Action Program (WRAP) [15]

DM, dry weight; CFU, colony forming units; MPN, most probable number; g, grams.

**Table 2:** Pathogen type, prevalence in cattle, temperature of storage, survival rates and diseases/symptoms caused in humans.

Pathogen Type	Prevalence (%)	Survival Rate (Days)	Storage Temperature (°C)	Diseases/Symptoms	References
*E. coli* O157:H7	16	10 to >100	5 to 30	Mild to bloody diarrhea, vomiting, hemolytic-uremic syndrome, hemorrhagic colitis	Olson [25] *Chekabab et al. [26]*
*Salmonella typhimurium, Salmonella dublin*	0 to 13	28 to 196	5 to 30	Salmonellosis	You et al. [27] *Martin [8]*
*Campylobacter* spp.	31.1	7 to 21	5 to 30	Campylobacteriosis, Guillain-Barre syndrome, reactive arthritis and postinfectious irritable bowel syndrome	Hakkinen et al. [28] *Olson [25]*
*Listeria monocytogenes*	24.4	168	<20	Listeriosis, flu-like Symptoms, vomiting, diarrhea, meningitis, septicemia, spontaneous abortions	Nightingale et al. [29] Nicholson et al. [30] *Vivante et al. [31]*
*Yersinia enterocolitica*	<1	10 to 100	5 to 30	Yersiniosis, diarrhea, lymphadenitis, pneumonia, abortions	Tirzui et al. [32] *Olson [25]*
*Cyptosporidium parvum*	1 to 100	28 to 56	5 to 30	Gastroenteritis and cyptosporidiosis	Olson [25] *Gerba and Smith [17]*
*Giardia lamblia*	10 to 100	7	5 to 30	Giardiasis (diarrhea and abdominal cramps)	Olson [25] Gerba and Smith [17]
